# Role of Temperature in Arsenic-Induced Antisurfactant Growth of GaN Microrods

**DOI:** 10.1021/acsomega.2c02777

**Published:** 2022-07-05

**Authors:** Paulina Ciechanowicz, Sandeep Gorantla, Monika Wełna, Agnieszka Pieniążek, Jarosław Serafińczuk, Bogdan Kowalski, Robert Kudrawiec, Detlef Hommel

**Affiliations:** † Łukasiewicz Research Network—PORT Polish Center for Technology Development, Wrocław 54-066, Poland; ‡ Faculty of Physics and Astronomy, University of Wrocław, Wrocław 50-137, Poland; § Department of Semiconductor Materials Engineering, Wrocław University of Science and Technology, Wrocław 50-370, Poland; ∥ Institute of Low Temperature and Structure Research, Polish Academy of Sciences, Wrocław 50-422, Poland; ⊥ Department of Nanometrology, Wroclaw University of Science and Technology, Janiszewskiego 11/17, Wroclaw 50-372, Poland; # Institute of Physics, Polish Academy of Sciences, Lotników 32/46, Warsaw 02-668, Poland

## Abstract

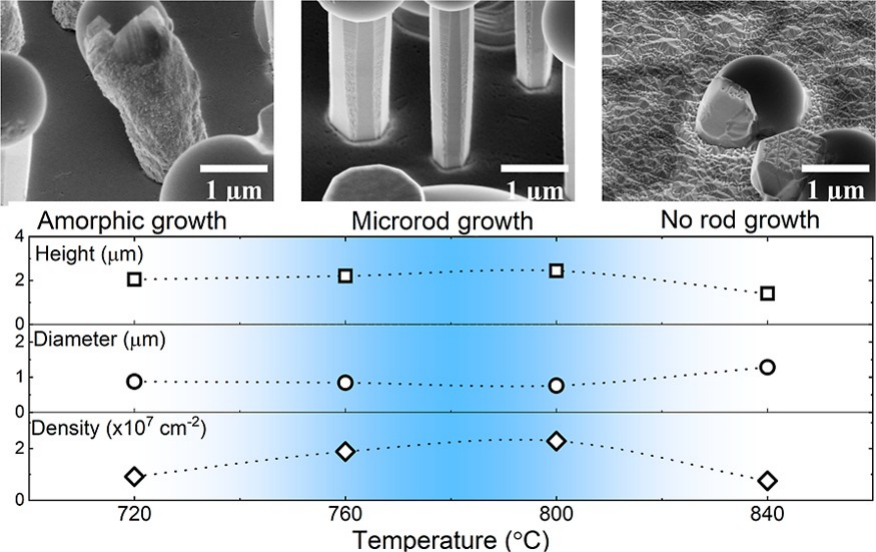

Due to the antisurfactant properties of arsenic atoms, the self-induced dodecagonal GaN microrods can be grown by molecular beam epitaxy (MBE) in Ga-rich conditions. Since temperature is a key parameter in MBE growth, the role of temperature in the growth of GaN microrods is investigated. The optimal growth temperature window for the formation of GaN microrods is observed to be between 760 and 800 °C. Lowering the temperature to 720 °C did not change the growth mechanism, but the population of irregular and amorphous microrods increased. On the other hand, increasing the growth temperature up to 880 °C interrupts the growth of GaN microrods, due to the re-evaporation of the gallium from the surface. The incorporation of As in GaN microrods is negligible, which is confirmed by X-ray diffraction and transmission electron microscopy. Moreover, the photoluminescence and cathodoluminescence characteristics typical for GaN are observed for individual GaN microrods, which additionally confirms that arsenic is not incorporated inside microrods. When the growth temperature is increased, the emission related to the band gap decreases in favor of the defect-related emission. This is typical for bulk GaN and attributed to an increase in the point defect concentration for GaN microrods grown at lower temperatures.

## Introduction

The growth of GaN-based vertical nano- and microstructures is nowadays a rapidly expanding field because of the unique properties of such structures in comparison to that of two-dimensional (2D) layers. Vertical structures are usually characterized by lower defect density and more effective strain relaxation compared to 2D layers.^1^ GaN nano- and micropillars have already been successfully used for application in microscale optoelectronics and photonics,^2−7^ high-power high-temperature electronic applications,^8,9^ and photocatalytic applications.^10−12^

There are many studies showing growth methods for GaN-based columnar structures such as nanorods,^2,13,14^ nanowires,^15^ micropillars,^16^ or microrods.^17,18^ Such structures are grown epitaxially by various methods including metal–organic chemical vapor deposition,^19−21^ chemical vapor transport,^22^ molecular beam epitaxy (MBE),^23−26^ and hydride vapor phase epitaxy.^27,28^

Recently, our studies showed that the growth of dodecagonal GaN microrods with dominating *a*-planes is possible by arsenic-induced vapor–liquid–solid (VLS)–MBE growth.^29^ It is well-known that critical parameters in all MBE growth processes are the III/V elements ratio and substrate temperature. Many studies showed that temperature has a vital influence on the crystal quality and surface roughness^30,31^ and on defect states and stress relaxation.^32^ MBE growth of GaN at high temperatures leads to 2D layer-by-layer growth and a smooth surface. It has also been shown that the surface roughness of *a*-plane GaN decreased with the increasing temperature, which can be explained with a larger diffusion length of atoms at higher growth temperatures.^33^ Moreover, it is worth noting that under N-rich conditions at very high growth temperatures, GaN starts to decompose, so growth can no be longer observed.^31,34,35^ Thus, the growth temperature has to be low enough to avoid gallium evaporation from the surface. On the other hand, too low temperature results in higher defect density, low mobility, and 3D island growth or even amorphous growth.^36−39^ Although temperature is a critical parameter in MBE growth, there are not many studies showing detailed analysis of change in crystal quality and optical parameters with temperature.

In our previous work, the growth parameters such as gallium and arsenic fluxes were determined to have a vital influence on the growth mode regime.^29^ It was shown that obtaining columnar growth is possible only in Ga-rich conditions under high arsenic overpressure. These growths were performed at an arbitrary chosen temperature of 800 °C, which is close to the optimal growth temperature of high-quality GaN layers.^40^ At this high temperature and in Ga-rich conditions, arsenic shows antisurfactant properties and is not built into the GaN matrix. Since temperature is known to have an impact on growth parameters in MBE, further studies in a wide temperature range are needed and would be interesting to perform. Moreover, regarding growth of GaNAs alloys, it has already been shown that with the decrease in growth temperature, As solubility in GaN increases.^41,42^ GaNAs alloys in the whole composition range can be obtained at a low temperature under N-rich conditions for MBE growth.^43^ From this point of view, investigating antisurfactant properties of arsenic in a wide temperature range is crucial to fully understand the VLS–MBE growth mode of GaN microrods. Such studies are reported in this paper. We focused on investigating the antisurfactant properties of arsenic during MBE growth, including its incorporation into GaN microrods and outside of the microrods.

## Results and Discussion

In order to study the temperature influence on the growth process, all other growth parameters were kept constant between processes. Samples were grown under a beam equivalent pressure of Ga equal to 4.3 × 10^–7^ mbar, a high arsenic overpressure above 1 × 10^–6^ mbar, and a nitrogen flux of 5 sccm with a plasma power of 500 W. According to our previous research, these parameters are optimal for the formation of GaN microrods.^29^ The growth temperature was varied in steps of 40 °C from 720 °C up to 880 °C.

**Figure 1:**
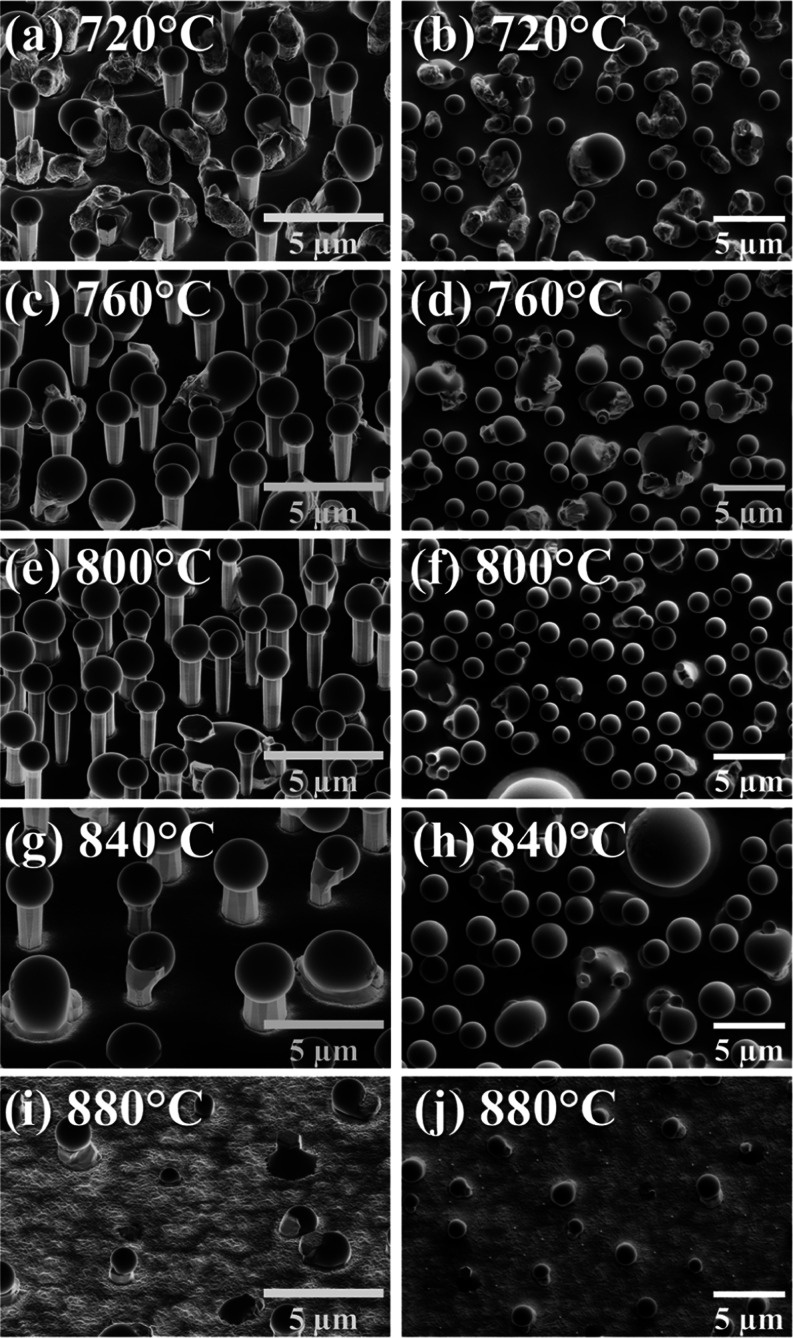
(a–j) SEM images of samples grown under different temperatures. Growth temperature for each sample is given in the image. For every pair of images, the left one shows the tilt view and the right one shows the top view.

**Figure 2:**
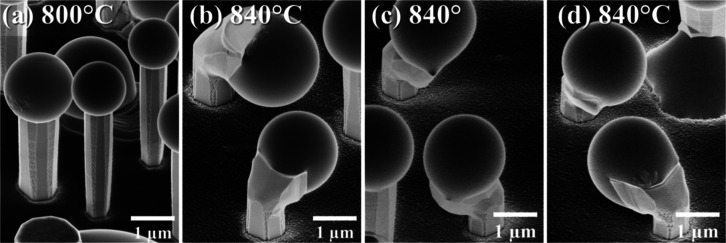
SEM images of individual GaN microrods grown at 800 °C (a) and 840 °C (b–d).

**Figure 3:**
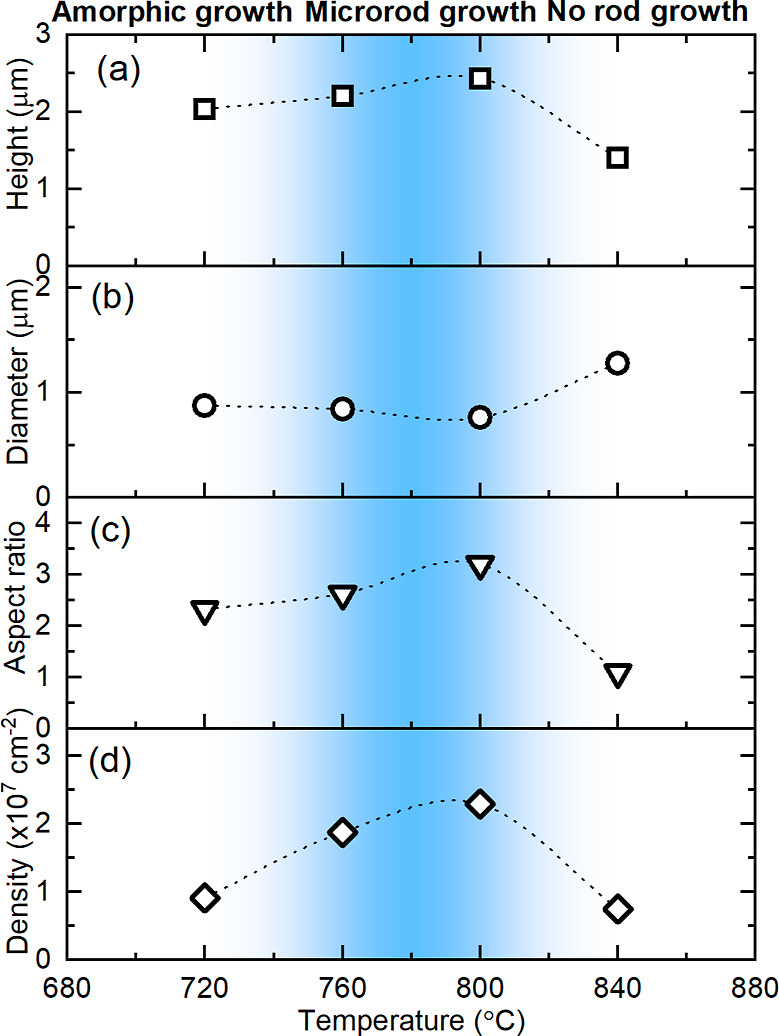
Dimensions of microrods (a,b), aspect ratio (c), and density (d) vs the growth temperature for samples obtained at different temperatures.

Figure 1 shows the scanning electron microscopy (SEM) images of five samples grown at different temperatures with the tilted and top views for each sample. In a wide temperature range of 720–840 °C, we observe that arsenic exhibits antisurfactant properties and leads to the formation of Ga droplets, which are catalysts for the growth of microrods (Figure 1a–h). At the lowest temperature, we observe two kinds of microrods: dodecagonal and amorphic-like. This indicates that the temperature of 720 °C is too low for microrod formation, which results in low mobility of incoming atoms. At a medium temperature range (760–800 °C), atom mobility increases and high-quality dodecagonal rods are visible with uniform geometry and dimensions. Going toward higher temperatures, a change in the morphology can be observed. At 840 °C, arsenic still works as an antisurfactant in Ga droplet formation, but some droplets start to melt and cause growth not in the vertical direction but at some angle. Figure 2 shows a magnification of GaN microrods grown at this temperature versus the representative regular GaN microrods obtained at the optimal growth temperature. At 880 °C, almost no droplets and rods are present since gallium is re-evaporating from the surface due to too high temperature. Therefore, Ga droplets are not formed and can no longer act as seeds for microrod growth. The surface in this sample is visibly more rough, which can be an indicator of GaN decomposition at high temperature under a nitrogen environment.^44^Figure 3 shows the relation between growth temperature and the height, diameter, aspect ratio, and planar density of microrods for samples grown in the temperature range 720–840 °C. The sample grown at 880 °C is not included since no rods could have been observed at this temperature. All the parameters are almost constant for samples grown at 720, 760, and 800 °C. This means that the growth rate is not affected by the temperature but is limited by one of the fluxes, Ga or N. Since the growth regime is Ga-rich, nitrogen seems to be the limiting factor. For samples grown at 720 °C, only high-quality rods were taken into account for the estimation of their density and dimensions. Thus, despite the very similar morphology of well-shaped rods, their density is much smaller than the density of rods in samples grown at higher temperatures. For the sample grown at 840 °C, the growth rate drops rapidly and the surface becomes rough probably due to the decomposition of GaN.

**Figure 4:**
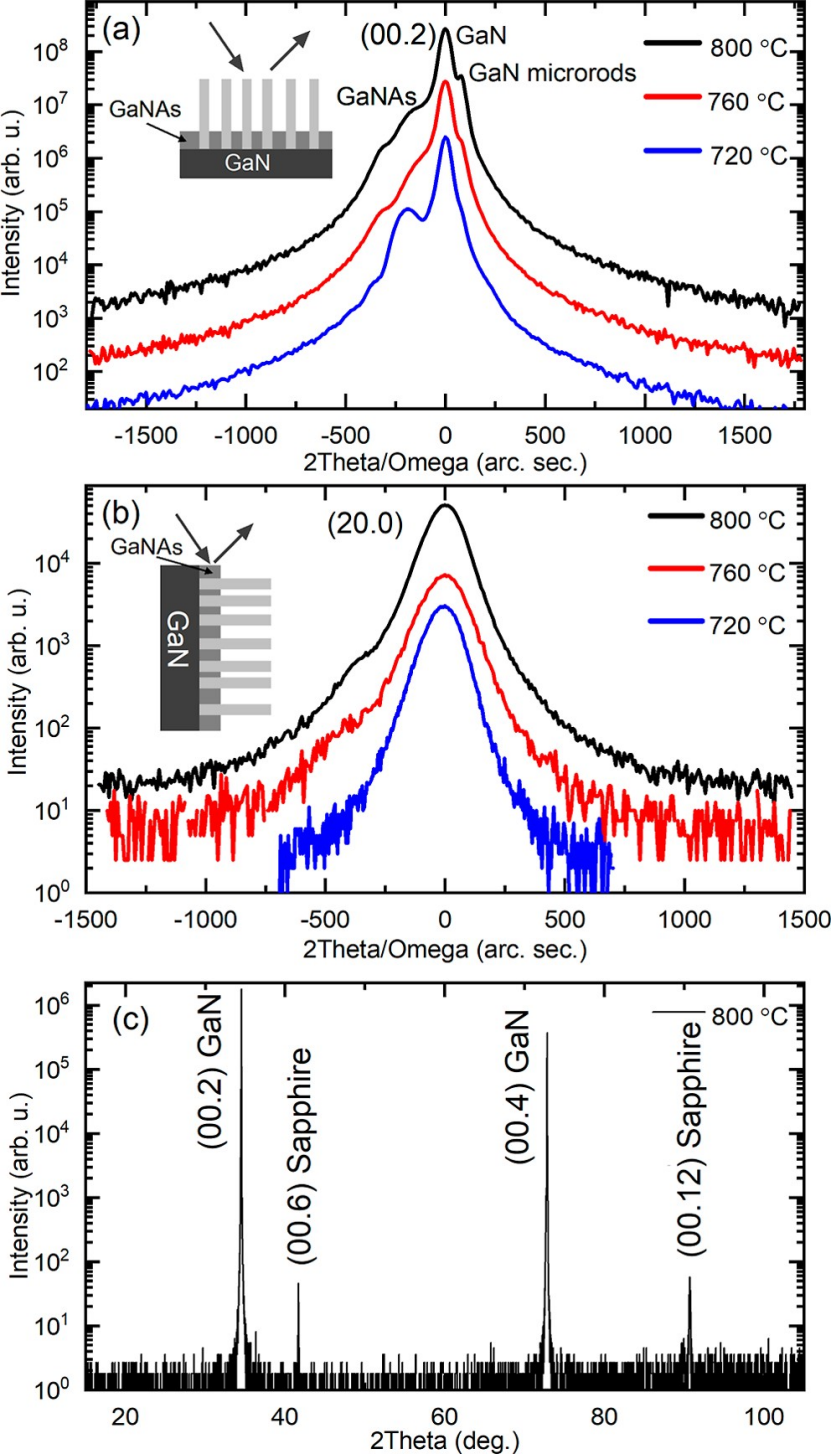
XRD diffraction curves for samples grown at 720, 760, and 800 °C obtained from the surface (a) and from the edge (b) of the sample. (c) Full-scale XRD pattern from the sample grown at 800 °C.

Based on the morphology of the samples, we can conclude that arsenic works as an antisurfactant in Ga-rich conditions up to the temperature of Ga re-evaporation. The temperature window for the formation of GaN microrods is quite broad, 720–840 °C, but the optimum temperature range can be chosen as 760–800 °C, which is shown in Figure 3 as the blue region. In conclusion, temperature is crucial in GaN microrod formation for two reasons. First, if the temperature is too low, precursor mobility is not sufficient to ensure high-quality crystal formation. On the other hand, high temperature prevents Ga droplet formation due to very high re-evaporation. Further experiments were performed to investigate arsenic incorporation into the microrods as it was observed that the growth temperature strongly influence As incorporation into GaNAs when the alloy is grown near stoichiometric conditions^42,43^ The microstructural properties of the microrods were characterized by X-ray diffraction (XRD) and scanning transmission electron microscopy high-angle annular dark field (STEM-HAADF). Figure 4 shows 2θ scans obtained from the sample surface and from the edge of the sample, as shown in the sketch in panels (a) and (b) for samples grown at 720, 760, and 800 °C. In the case of measurements from the sample surface (Figure 4a), the reflections coming from the plane (00.2) (the plane parallel to the sample surface) is observed. The strongest peak is attributed to the GaN template. In addition, a weak peak is observed on the left side of the GaN peak and is attributed to the incorporation of As into GaN. As is shown later, As is not incorporated into microrods, and therefore, this peak is assigned to the GaNAs layer that grows between columns. The layer is thin compared to the height of the microrods, but its thickness is sufficient to observe the GaNAs layer in XRD. The arsenic concentration in this layer is estimated to be 0.3% for the sample grown at 720 °C and drops to 0.2% for the sample grown at 800 °C. Detailed analysis of the GaNAs layer between rods will be discussed elsewhere since it has no impact on rods analysis. In the case of the XRD scan from the sample edge (Figure 4b), that is, reflections coming from the plane (20.0) (the crystal plane perpendicular to the sample surface), the GaNAs layer is not observed. This experimental configuration is not favorable to probing a thin layer that is perpendicular to the edge from which the 2θ scan is performed. Moreover, this scan probes the lattice constant *a*, which for a thin GaNAs layer should be the same as that in GaN. In this case, the (20.0) peak from GaN microrods overlaps with the (20.0) peak from the GaN template. The same is observed for the 2θ scan from the sample surface, that is, panel (a), where the main peak overlaps with the (00.2) peak from GaN microrods, whose intensity is weaker due to the smaller amount of material probed by the X-ray beam. However, for samples grown at higher temperatures (760 and 800 °C), an additional peak is resolved on the right side of the main peak (Figure 4a). This feature is attributed to GaN microrods, and its angle position means that the lattice constant *c* in GaN microrods is smaller than that in the GaN template. The difference in the lattice constant has been estimated to be 0.0031 Å, that is, ∼0.06%, and is due to no residual strain in GaN microrods. In general, a similar feature could be observed in the 2θ scan from the sample edge, but the broadening of the (20.0) peak is too large in this case. Figure 4c shows the complete 2θ scan of the sample grown at 800 °C. All reflections have been indexed according to hexagonal wurtzite GaN and sapphire. Observed peaks in this pattern confirm the *c* orientation of both the GaN template and microrods.

**Figure 5:**
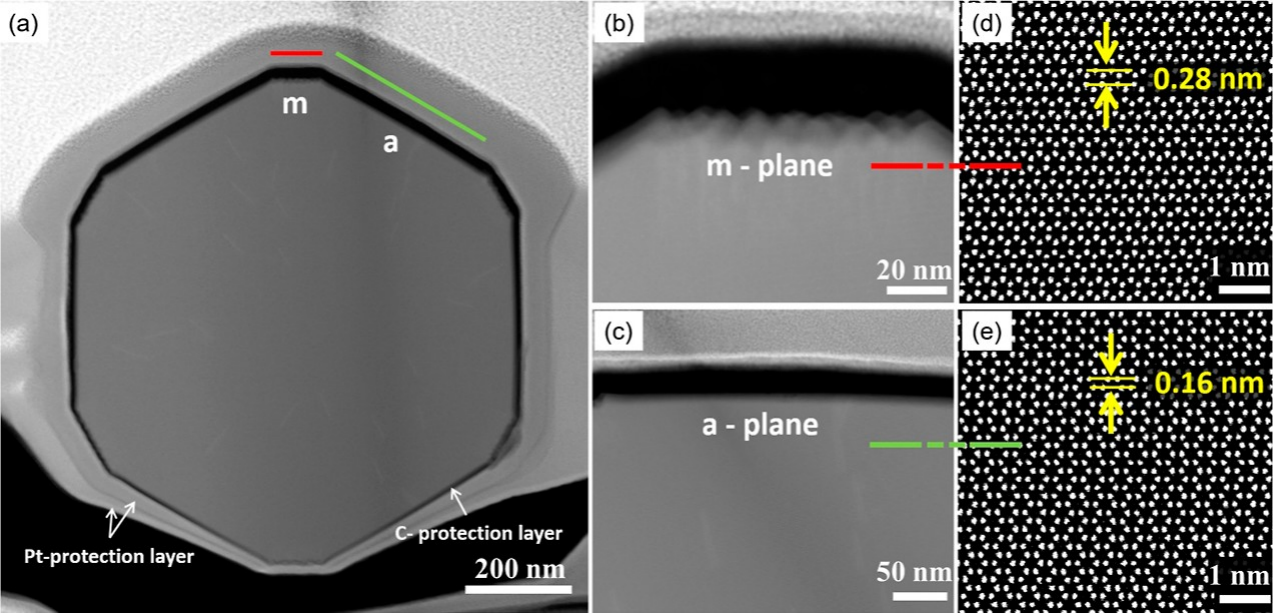
(a) STEM-HAADF characterization of GaN microrod growns at 720 °C in the horizontal cross-section. (a) Low-magnification STEM-HAADF image of microrods, along its horizontal cross-section, clearly shows 12 faceted outer walls. (b,c) higher-magnification images of individual *m* and *a* plane facets, respectively. (d,e) the HRSTEM-HAADF images of the microrod’s GaN crystal structure parallel to *m* and *a* plane facets, respectively, where the overlaying markers, spanning over (b,d) and (c,e), provide a guide to the eye. The *d*-spacings in (d,e) corresponding to (1–100) and (11–20) planes, respectively, are shown. The images (d,e) are processed using *ImageJ* Otsu auto threshold adjustment (raw images are shown in the Supporting Information).

**Figure 6:**
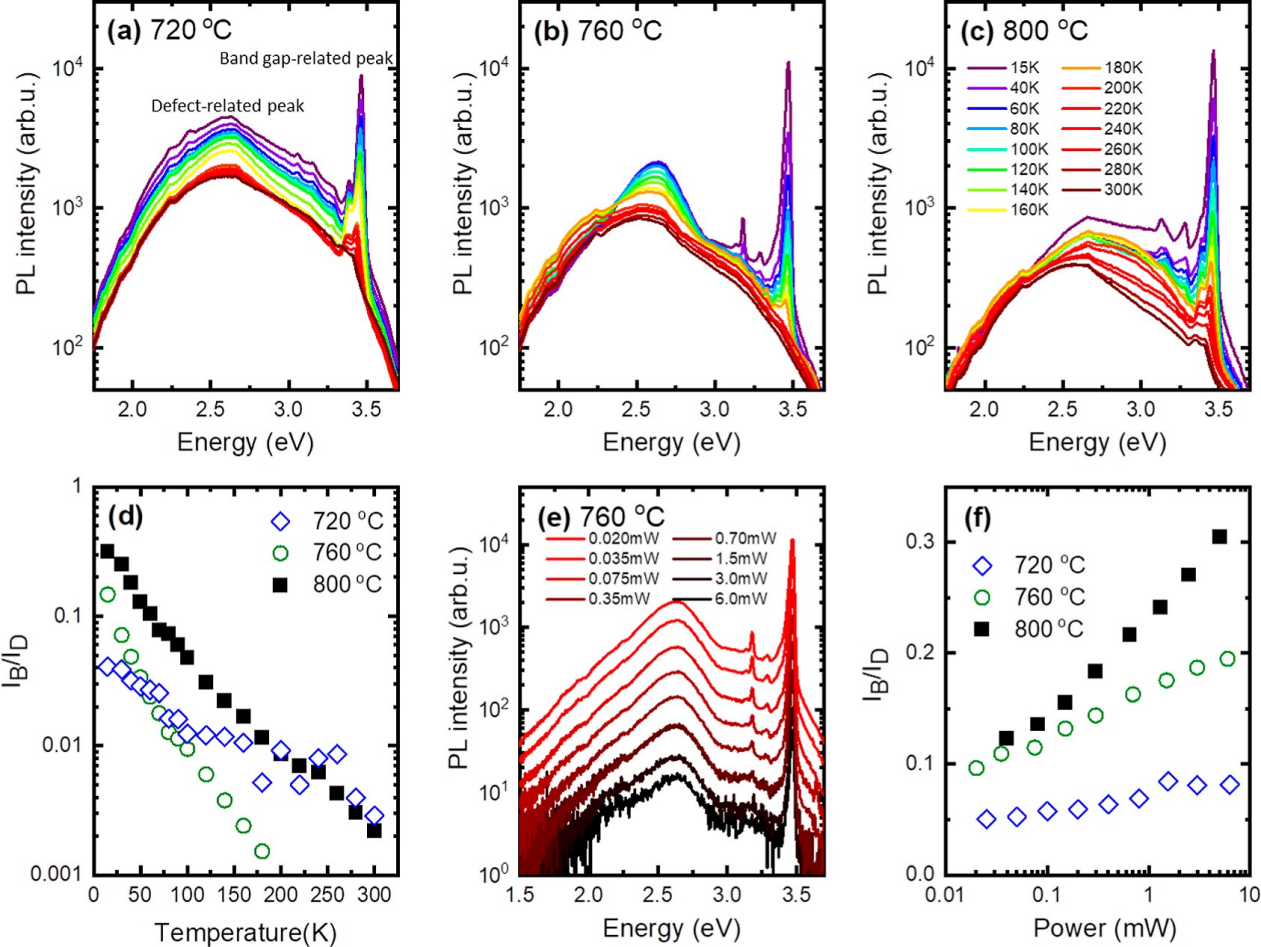
PL temperature dependence spectra for samples grown at 720 (a), 760 (b), and 800 °C (c). (d) Ratio of band gap-related emission and defect-related emission. (e) PL spectra measured at different powers at 20 K for the sample grown at 760 °C. (f) Power dependence of the ratio of band gap-related emission and defect-related emission at 20 K for samples obtained at different temperatures.

The microstructure of the outer facets of the microrods grown at 720 °C was further analyzed using the STEM-HAAADF technique. For this purpose, the horizontal cross-section of the microrod perpendicular to its length axis, that is, the growth direction, was investigated. The growth direction was determined to be the [0001] direction of the GaN wurtzite structure in our previous work of similar microrods.^29^Figure 5a shows the overview STEM-HAADF image of the microrod cross-section, and the 12 faceted outer walls can be clearly seen here. Figure 5b,c shows the representative magnified images of individual shorter and longer facets of the microrod. To determine the planes corresponding to the shorter and longer facets of the microrod, HRSTEM-HAADF imaging was performed to image the atomic columns of Ga in the GaN structure at each of the two different facets. Figure 5d shows the arrangement of the Ga atomic columns parallel to the orientation of the shorter facets in Figure 5b, while Figure 5e shows the Ga atomic columns parallel to the orientation of the longer facets in Figure 5c; an overlaying marker is placed to serve as the eye guide of this orientational relationship between the corresponding figures. Based on the orientation of Ga atomic columns with respect to the unit cell of the GaN wurtzite structure, as seen in Figure 5d,e, and using the measured *d*-spacing of atomic planes, that is, 0.28 and 0.16 nm, the obtained numbers are typical for bulk GaN (ICSD database #157398). These observations are consistent with the result obtained from the microrod grown at 800 °C.^29^ The longer, smooth edge corresponds to *a*-planes of the GaN structure and the shorter rough edges correspond to *m*-planes. Moreover, this result proves that arsenic is not built into the structure of the microrod, which confirms the hypothesis that the signal observed from XRD measurements assigned to the GaNAs layer originates from layers growing between rods and not rods itself. The TEM research carried out for the microrods obtained at 800 °C^29^ and presented in this article for the sample grown at 720 °C clearly shows that the incorporation of As inside GaN microrods is negligible. Therefore, it can be assumed that in the entire temperature range of 720–800 °C, arsenic works as an antisurfactant and does not build up inside the microrods. Figure 6 shows the temperature-dependent photoluminescence (PL) spectra obtained from microrods grown at 720, 760, and 800 °C (Figure 6a–c). In order to avoid the signal from the GaN template, microrods have been scratched off from the GaN substrate and dispersed onto a sapphire substrate. For the three samples, PL spectra typical for GaN are observed.^45−47^ Two emission bands are observed, as marked in the graph: the sharp one is the GaN band gap-related emission and the second one is a wide yellow band from defects at lower energies around 2.5 eV. Due to the fact that the surface is a dead layer for PL, we cannot exclude a small amount of arsenic on the surface of the microrods, but we can certainly say that the inside of the microrods is pure GaN because the emission typical for GaN would not be observed otherwise. The observed differences are attributed to the different concentrations of point defects and not the presence of arsenic inside the GaN rods because even a small amount of arsenic would have reduced the energy gap down to ∼2.8 eV.^40,48^ The direct comparison of PL intensities for the three samples is difficult because of the different amounts of the material, which is excited in these measurements performed on microrods transferred onto sapphire substrates. Therefore, to compare the optical quality of the studied samples, the ratio of band gap-related emission (*I*_B_) and defect-related emission (*I*_D_) was analyzed (Figure 6d). This ratio varies very significantly with temperature and is in favor of the band gap-related emission for the sample grown at 800 °C. In addition, the power-dependent PL spectra were measured at a low temperature, and the *I*_B_/*I*_D_ ratio was analyzed (Figure 6f). From this analysis, it is concluded that the highest *I*_B_/*I*_D_ ratio, that is, the highest optical quality, is observed for samples grown at higher temperatures, that is, at 760 and 800 °C.

**Figure 7:**
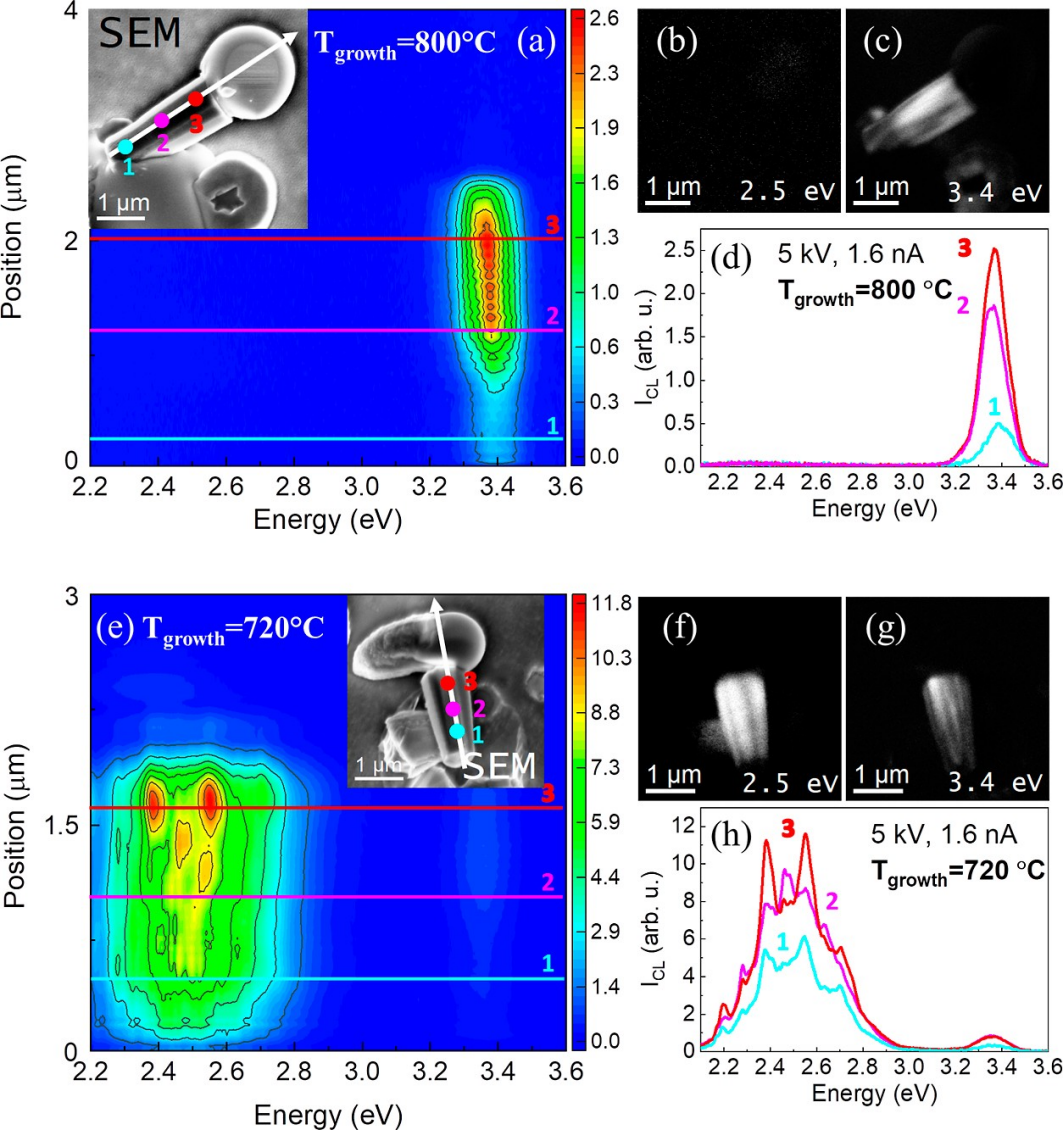
CL line scans acquired along the *z*-axis of individual microrods grown at 800 (a) and 720 °C (e). Monochromatic CL maps of microrods in the inset in (a) obtained at 2.5 (b) and 3.4 eV (c). (d) CL spectra obtained from places marked in the inset in (a) as (1), (2), and (3). Monochromatic CL maps of microrods in the inset in (e) obtained at 2.5 eV (f) and 3.4 eV (g). (h) CL spectra obtained from places marked in the inset in (e) as (1), (2), and (3).

The optical properties of the GaN microrods were also studied by spectrally and spatially resolved cathodoluminescence (CL) spectroscopy and imaging (Figure 7). The submicron spatial resolution of this method allows us to take the monochromatic CL map at the defined wavelength from selected regions of the sample. GaN microrods obtained at 720 and 800 °C were selected for this study. They were investigated in the side view regime, with the electron beam parallel to the *c*-plane of the structure.

The inset in Figure 7a illustrates the electron beam path orientation (marked with the white arrow in the SEM image) with respect to the structure, from the base to the top. Figure 7a shows the CL line scan acquired along the *z*-axis of an individual microrod grown at 800 °C. A strong band gap-related emission located at 3.4 eV is clearly visible on this map as well as in Figure 7d, where CL spectra obtained from places marked in the inset as (1), (2), and (3) are plotted. Defect-related emission, which is usually observed at 2.5 eV, is negligible in these spectra. Monochromatic CL maps obtained at 2.5 eV (Figure 7b) and 3.4 eV (Figure 7c) confirm the low concentration of defects and strong band gap-related emission, respectively. It is worth noting that the defect-related emission was observed for this sample in PL analysis, but excitation conditions in CL measurements are much stronger, and therefore, the defect-related emission is saturated and much weaker than the band-gap related emission. In the case of microrods grown at 720 °C, the defect-related emission prevails in both the CL line scan (Figure 7e) and monochromatic CL maps (Figure 7f,g), indicating a higher concentration of defects in this microrod. In addition, CL spectra presented in Figure 7h confirm strong defect-related emission centered at 2.5 eV.

Summarizing CL and PL measurements performed on GaN microrods grown at different temperatures, we can conclude that no emission, which could be attributed to GaNAs, that is, an emission at 2.8 eV,^40,48^ is visible in these spectra. The observed spectra are typical for GaN.^45−47^ They change with the growth temperatures because of the different contribution of the defect-related emission and the band-gap related emission. The latter is weaker for microrods grown at lower temperatures because of the stronger nonradiative recombination.

## Conclusions

It is concluded that arsenic works as an antisurfactant in the studied temperature range, but at too low growth temperatures, below 740 °C, the atoms lose mobility and amorphous-like microrods appear, and at too high growth temperatures, gallium evaporates from the surface and gallium-rich conditions are no longer assured. No significant As incorporation into GaN microrods was confirmed by structural and optical study that is consistent with the antisurfactant character of this component in the MBE process in Ga-rich conditions. The optimal window growth temperature for the deposition of high-quality GaN microrods was established to be 760–800 °C. Microrods grown in these conditions show a well-shaped morphology, high crystal quality, and good optical properties with weak defect-related emission. Outside this temperature window, the crystal quality and optical quality deteriorate both at higher and lower temperatures.

## Methods

### Molecular Beam Epitaxy

Samples were grown by plasma-assisted MBE in a dual chamber MBE system (Scienta Omicron GmbH). A Knudsen effusion cell was used as a source of metallic gallium. Arsenic in the form of dimers (As_2_) was produced using an arsenic-valved cracker source. The nitrogen RF plasma source was used for generating active nitrogen. As a substrate, we used sapphire with a GaN layer grown by metalorganic vapor-phase epitaxy. The templates were back-coated with Ti to provide heat distribution. The substrate temperature during the process was measured using a thermocouple. Real growth temperature is about 120 °C lower than thermocouple reading.

### Scanning Electron Microscopy

Samples were imaged using FEI Helios NanoLab 660. A 2 kV acceleration voltage was used to observe the morphology of each structure.

### High-Resolution X-ray Diffractometry

The Empyrean high-resolution X-ray diffractometer equipped with a hybrid monochromator in the incident beam path and a Pixcel3D detector, and a double crystal analyzer in the diffracted beam optics was used in the measurements of diffraction curves. In addition, the measurements used an X-ray tube generating the beam Cu k_α1_ = 1.540597 Å, while the goniometer allows for measurements with the angular resolution of 2θ = 0.0002°. The X-ray tube power was set to 40 kV and 40 mA, while the measurements were performed under normal conditions.

### TEM Specimen Focused Ion-Beam Lamella Preparation

Initially, the surface of a sample with microrods was manually scratched to force some microrods to fall and lay flat along their length axis. The sample was then coated with carbon, using a c-thread sputter coater, with a thickness of ∼30 nm. Then, with the aid of SEM imaging, an individual microrod, lying flat on the surface, was selected for TEM specimen preparation using a ThermoFisher Scientific Helios NanoLab 450HP dual-beam SEM system equipped with a Ga^+^ ion column. TEM lamella is prepared using the typical focused ion beam (FIB) milling methodology. In brief, in FIB-SEM, initially, an electron beam-induced platinum (Pt) layer with a thickness of 300 nm and then ion-beam-induced Pt layer with a thickness of 3 μm are deposited as additional protection layers over the microrod. This was followed by ion-beam milling of a section of the microrod with a 2 μm width. The milled section is then lifted out through the nano manipulator and welded to a copper TEM half grid with posts. This section was then gradually thinned to electron transparency in steps, starting from 30 kV and 2.5 nA current down to 30 kV and 83 pA until a thickness of 100 nm is achieved. After this step, final thinning of the lamella is performed in gradual steps from 5 kV and 41 pA down to 1 kV and 28 pA. The final polishing step was performed with 1 kV and 28 pA current for 30 s on both sides of the lamella with the electron beam blanked; this helps in removing any FIB-induced surface amorphous layer and Pt surface contamination.

### Scanning Transmission Electron Microscopy

The ThermoFisher Scientific Titan 60-300 cubed S/TEM microscope equipped with a high-brightness X-FEG electrons emitter, a Wien-filter monochromator, an image Cs-corrector, a DCOR probe Cs-corrector, a ChemiSTEM super-X EDS 4-detectors system, and a continuum electron energy loss spectroscopy spectrometer was used for the STEM characterization of the microrods. The microscope was operated at 300 kV accelerating voltage. STEM-HAADF imaging was performed with a probe current of ∼ 80 pA and a beam convergence angle of 21.4 mrad, and the HAADF detector collection angles were in the range 50.5–200 mrad.

### Photoluminescence Spectroscopy

For PL measurements, samples were mounted in a closed-circle refrigerator, allowing measurements from 10 K up to 360 K. The samples were excited by a 325 nm line from a Kimmon HeCd Laser. The PL signal was detected using an Avantes spectrometer equipped with a one-stage thermoelectrically cooled CCD array detector.

### Cathodoluminescence Spectroscopy

CL spectra were measured using a scanning electron microscope Hitachi SU-70 that was equipped with the Gatan Mono CL3 system and a grating with 300 lines per mm. The experiment was performed at 300 K under excitation conditions of an accelerating voltage of 5 kV and an electron beam current of 1.6 nA.

## Supporting Information Available

Additional experimental details including the unprocessed HRSTEM-HAADF images and EDS map and point spectra of the microrod (PDF)
