# Supplementary material for: Role of Temperature in Arsenic-Induced Antisurfactant Growth of GaN Microrods
Source: ACS Omega. 2022 Jul 5;7(28):24777–84. doi: 10.1021/acsomega.2c02777 (PMC9301637; doi:10.1021/acsomega.2c02777)
Supplement: Supplementary file 1 — ao2c02777_si_001.pdf [file ao2c02777_si_001.pdf]

The Role of Temperature in Arsenic-Induced Antisurfactant Growth of GaN Microrods

Paulina Ciechanowicz, [†,‡] Sandeep Gorantla, [†] Monika Wełna,[§] Agnieszka Pieniążek,[§] Jarosław Serafińczuk,[†, ⊥] Bogdan Kowalski,[f] Robert Kudrawiec,[†,§] Detlef Hommel[†,‡]

[†]Łukasiewicz Research Network – PORT Polish Center for Technology Development, Wrocław, Poland

[‡]Faculty of Physics and Astronomy, University of Wrocław, Wrocław, Poland

[§]Department of Semiconductor Materials Engineering, Wrocław University of Science and Technology, Wrocław, Poland

[∥]Institute of Low Temperature and Structure Research, Polish Academy of Sciences, Wrocław, Poland.

[⊥]Department of Nanometrology, Wroclaw University of Science and Technology, Janiszewskiego 11/17, 50-372 Wroclaw, Poland

[f]Institute of Physics, Polish Academy of Sciences, Lotników 32/46 ,02-668 Warsaw, Poland

Supplementary Figures

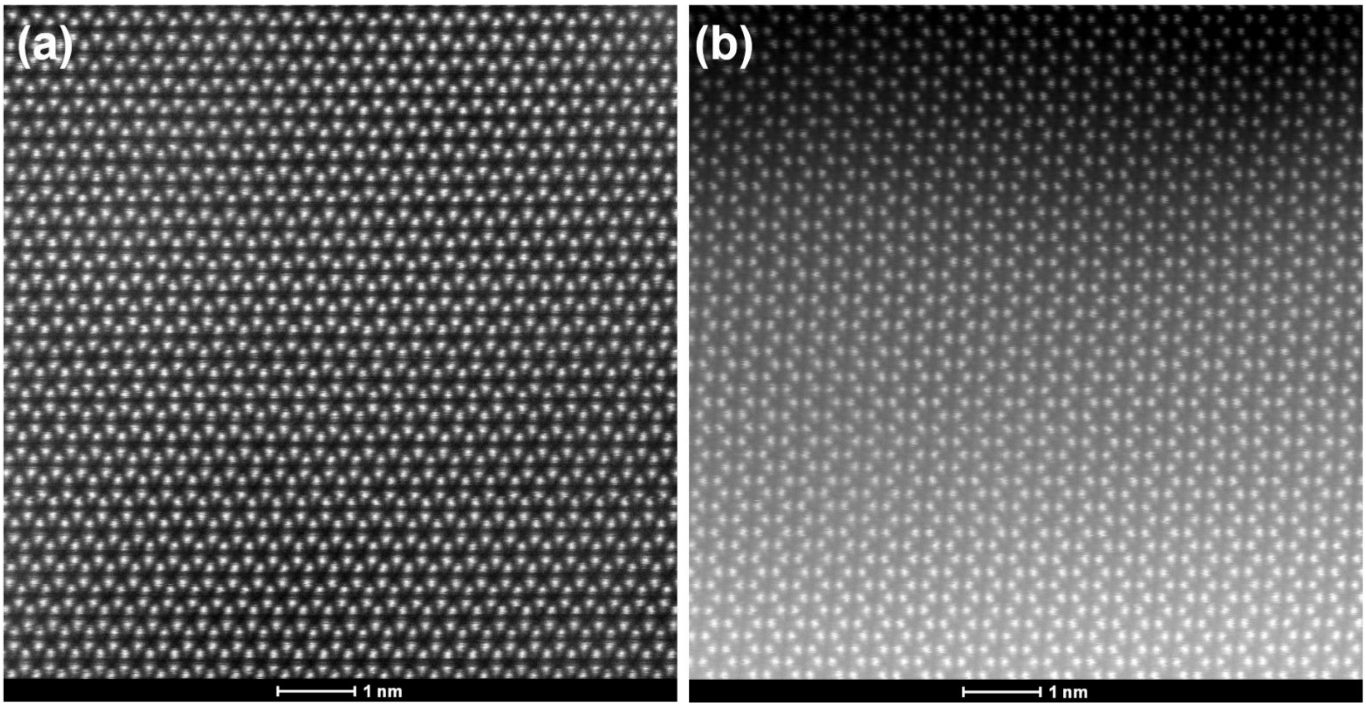

Figure S1. The unprocessed HRSTEM-HAADF images. (a) corresponds to main text figure 5(d) and (b) corresponds to main text figure 5(e).

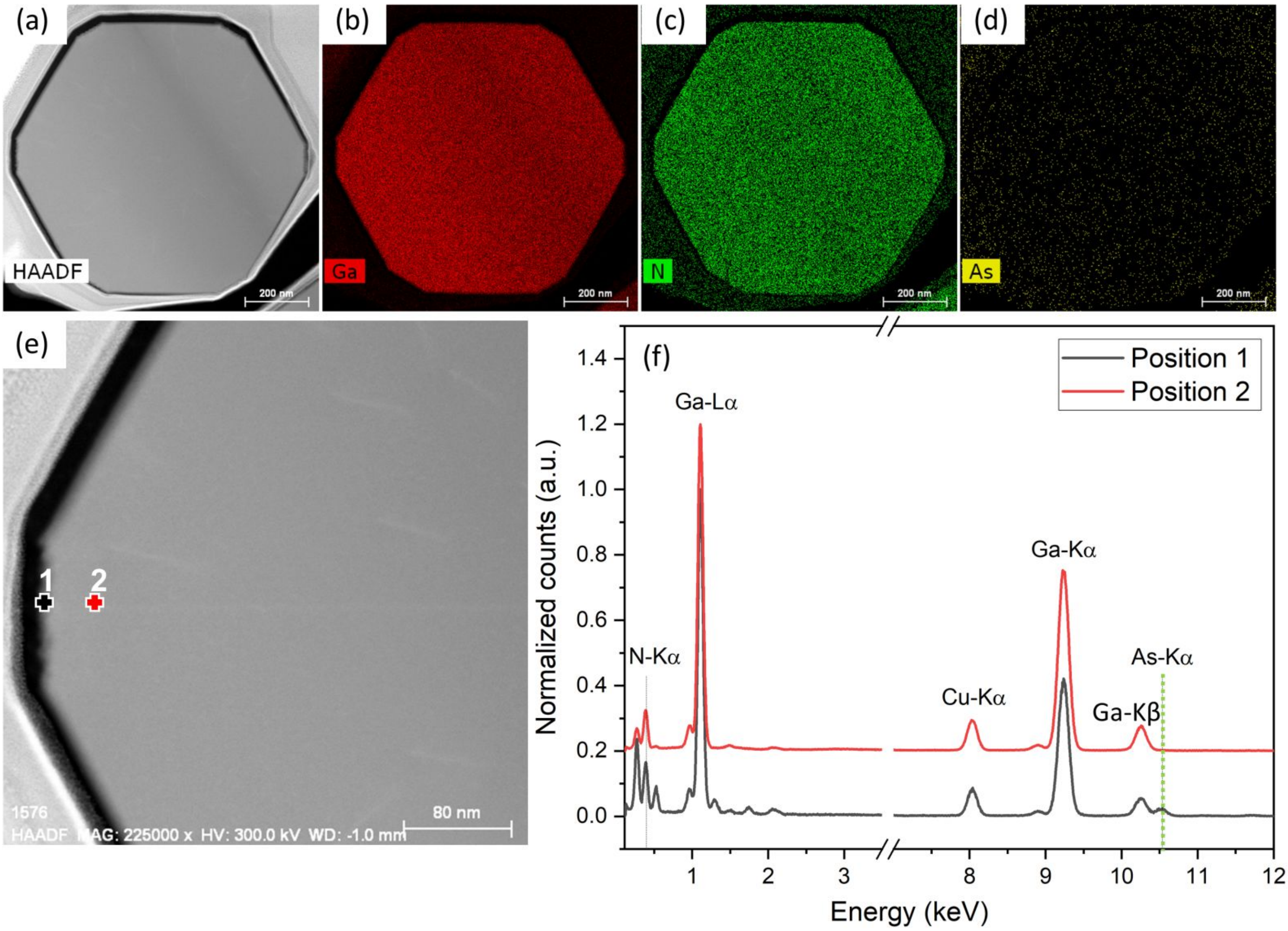

Figure S2. (a) STEM-HAADF image of a microrod horizontal cross-section, (b) - (d) corresponding X-ray EDS spectrum image elemental maps, (d) shows that signal-to noise ratio in As-Kα map is too weak; (e) STEM-HAADF image of an edge region of the microrod with EDS point acquisition positions 1, 2 marked, (f) the corresponding comparative EDS spectra from positions 1 and 2, it clearly shows that As-Kα peak was detected at microrod edge position and is absent inside the microrod bulk.
